# Optimization of ultrasound-assisted extraction of naturally occurring glucosinolates from by-products of *Camelina sativa* L. and their effect on human colorectal cancer cell line

**DOI:** 10.3389/fnut.2022.901944

**Published:** 2022-07-22

**Authors:** Stefania Pagliari, Chiara Maria Giustra, Chiara Magoni, Rita Celano, Paola Fusi, Matilde Forcella, Grazia Sacco, Davide Panzeri, Luca Campone, Massimo Labra

**Affiliations:** ^1^Department of Biotechnology and Biosciences, University of Milano-Bicocca, Milan, Italy; ^2^Department of Pharmacy, University of Salerno, Fisciano, Italy

**Keywords:** *Camelina sativa* L., ultrasound-assisted extraction (USAE), experimental design optimization, human colorectal cancer cell line, recovery bioactive compounds, glucosinolates derivatives, food by-products

## Abstract

The food waste generated by small and medium agro-industrial enterprises requires appropriate management and valorization in order to decrease environmental problems and recover high-value products, respectively. In this study, the *Camelina sativa* seed by-product was used as a source of glucosinolates. To begin, the chemical profile of the extract obtained using an international organization for standardization (ISO) procedure was determined by UPLC-HRMS/MS analysis. In addition, an extraction method based on ultrasound-assisted extraction was developed as an alternative and green method to recover glucosinolates. Main parameters that affect extraction efficiency were optimized using a response surface design. Under optimized conditions, the extract showed an improvement in extraction yield with a reduction in organic solvent amount compared to those obtained using the ISO procedure. Finally, the extract obtained with the ultrasound-assisted method was purified, tested on human colorectal cancer cell lines, and showed promising results.

## Introduction

Several epidemiological studies suggest a relationship between cruciferous vegetable intake and risk of several types of cancer. Higher intakes of cruciferous vegetables (more than three servings per week) have been associated with significant reductions in the risk of lung, stomach, and colorectal cancers, and with less consistent reductions in the risk of prostatic, endometrial, and ovarian cancers (1–4). Recent studies indicate that glucosinolates and their breakdown products including indoles and isothiocyanates have a beneficial health effect and may contribute to reduction of neurodegenerative and cardiovascular diseases when taken as part of the diet [(5–7); Le (8, 9)]. Glucosinolates (GLSs) are secondary metabolites produced by cruciferous plants. They accumulate at a high concentration in many species belonging to the Brassicaceae family. Nowadays more than 120 different glucosinolates have been identified in many plants such as mustard, cabbage, cauliflower, broccoli, and radish. The composition and concentration of GLSs have been shown to vary from one species to another and in a single variety depending on plant environment, crop conditions, age, and health (10–12). GLSs play an important role in plant protection (13, 14). In fact, these compounds remainin active unless they interact with an enzyme called myrosinase, which converts them into glucose and aglycones first and then into other molecules such as nitriles or isothiocyanates (15). The glucosinolate-myrosinase system is used as defense against the aggression of external plants, and for these reasons GLSs are also used as natural pesticide and biofumigation agents (16, 17). Different chemical and biological properties are the reason why these plant secondary metabolites attract the attention of several researchers (18). *Camelina sativa* L. is one of the plants in which GLSs are found. *C. sativa* appears to be an interesting agricultural crop because of its good oil yield with an omega-3 fatty acid content, which makes it a promising alternative source of essential fatty acids (19). *C. sativa* seed-press cake (PC) represents a co-product of a food chain particularly rich in interesting compounds such as glucosinolates, which could be used in the pharmaceutical, cosmetics, and food industries and whose valorization makes the entire supply chain environmentally and economically sustainable (19). However, in order to exploit *Camelina sativa* PC as a source of bioactive compounds, it is necessary to develop adequate extraction methods to reduce time, cost, and environmental pollution (20). Current GLS extraction methods involve several time-consuming and potentially hazardous steps. These steps are lyophilization, tissue disruption, and a long and laborious extraction protocol involving double extraction with boiling aqueous methanol.

In this study, a green extraction procedure was developed for recovery of glucosinolate compounds from by-products of *C. sativa* seeds. Initially, the international standard method ISO9167-1 (21) usually used for GLS extraction in order to obtain a reference extract characterized by ultra-pressure liquid chromatography (UPLC) coupled with a high-resolution mass spectrometry (HRMS) detector. After the chemical characterization, an extraction method based on ultrasound assisted (USA) technology using green solvents (water and ethanol) was developed. The main parameters of ultrasound-assisted solid liquid extraction (USAE) were carefully optimized using an experimental design to improve the extraction efficiency and reduce the consumption of organic solvents. Under optimized extraction conditions, the developed method demonstrated better efficiency than those obtained using the ISO procedure. Furthermore, a rapid procedure based on solid phase extraction (SPE) was applied on the USA extract to purify and concentrate glucosinolate compounds. Finally, the anticancer activity of the purified extract was measured *in vitro* on human colorectal cancer cell lines by viability assay to evaluate putative nutraceutical properties. Enriched glucosinolate fractions displayed selective cytotoxic activities against tumor cell lines but not against healthy lines and showed promising results for future studies.

## Materials and methods

### Standards and materials

MS-grade solvents used for UPLC analysis, acetonitrile (MeCN) water (H_2_O), and formic acid (HCOOH), were provided by Romil (Cambridge, United States); analytical-grade solvents methanol (MeOH) and ethanol (EtOH) were supplied by Sigma-Aldrich (Milan, Italy). Water was purified using a Milli-Q system (Millipore, Bedford, United States). Acetic acid (Sigma-Aldrich) and ammonium hydroxide solution were provided by Sigma-Aldrich (Milan, Italy). Glucoarabinin potassium salt, glucocamelinin potassium salt, and homoglucocamelinin potassium salt were purchased from Extrasynthese (Lyon, France). Stock standard solutions (1 mg ml^–1^) of each compound were prepared using methanol and stored at 4°C. Diluted solutions and mixtures were made in MeOH:H_2_O 1:1, (v:v).

### Samples

*Camelina sativa* PC was supplied by FlaNat Research srl (Milan, Italy). After cold oil extraction, the PC by-product was immediately finely blended using a knife mill, Grindomix GM-200 (Restek GmbH, Germany) operated at 6,000 rpm for about 30 short cycles of approximately 15 s each to prevent the samples from heating. The ground samples were sieved through a test sieve in the range of 300–600 μm to obtain a powder with homogeneous particle size distribution and stored in the dark at -80°C in polyethylene bags until analysis.

### Optimization of ultrasound-assisted extraction

Extraction of GLS compounds from PC was performed by ultrasound-assisted solid liquid extraction (USAE). For each extraction, 1 g of finely ground sample was placed in a 50-ml polypropylene tube, and an extraction solvent was added to the sample. Then, the tube was gently shaken by hands for a few seconds and immersed in an ultrasonic bath (frequency 35 kHz; power 60–120 W; Sonorex TK 52; Bandelin electronic, Berlin, Germany). During the extraction, the temperature of the water bath was continuously monitored and adjusted to maintain a constant temperature of 30°C. At the end of each extraction cycle, lasting 5 min for each, the samples were centrifuged (ALC centrifuge PK 120; Thermo Electro Corporation, San Jose, CA, United States) at 19.8 g. The supernatant was collected with a Pasteur pipette, filtered (Whatman No. 1 filter), and analyzed by UPLC-HRMS. To select the best extraction conditions, a central composite experimental design (CCD) was performed using Statgraphic Centurion XVI Version 16.1 (Rockville, MD, United States). The effect of four independent factors on extraction efficiency and the total amount of EtOH were studied through an experimental design. The range for each factor was selected by preliminary experiments. In particular, a response surface Box-Behnken design 2-factor interaction was carried out considering three variables at three different levels (low, medium, and high): solvent volume (vol) at 5, 10, and 15 ml; number of cycles (n°) 2, 3, and 4; and composition of solvent (EtOH%) 40, 60, and 80%. Four response variables were individually considered in the optimization of the extraction conditions: the extraction yield of each GLS was expressed as μg g^–1^of dry matter (ug/g DM) and total ethanol used (ml). A total of 16 experiments (16 points of the factorial design, 4 center points, and 6 freedom degrees) were carried out in a randomized run. Optimal experimental conditions that independently maximized extraction efficiency and minimized the total amount of EtOH used were obtained from a fitted model. Analysis of variance (ANOVA) was conducted to evaluate the statistical significance of independent variable contributions and their first-order interactions. The experimental matrix design, with the experimental conditions of each independent variables, and the results of experimental GLS extraction yield (μg/gDM) and total EtOH used (ml) from 16 selected combinations of the independent variables, are reported in Table 1.

**TABLE 1:** Experimental conditions of the response surface design and experimental values of the response variables.

	Independent variables			Response variables			
Experimental condition	EtOH (%)	Volume (mL)	Cycles (n°)	Glucoarabinin (μg/gDM)	Glucocamelinin (μg/gDM)	Homoglucocamelinin (μg/gDM)	Tot EtOH (mL)
1	40	5	3	493	1047	215	6
2	60	5	2	911	1984	387	6
3	80	10	2	832	1900	380	16
4	40	10	4	264	443	178	16
5	80	5	3	981	2234	428	12
6	80	10	4	1268	2981	568	32
7	80	15	3	1417	3240	631	36
8	40	10	2	526	1181	222	8
9	60	15	2	1304	3046	562	18
10	60	10	3	1166	2722	501	18
11	60	10	3	1221	2773	510	18
12	60	10	3	1269	2855	532	18
13	40	15	3	274	408	183	18
14	60	10	3	1142	2649	490	18
15	60	5	4	1180	2553	463	12
16	60	15	4	1354	2910	601	36

### Purification of glucosinolates by solid-phase extraction

A solid-phase extraction procedure was developed in order to obtain an extract rich in GLSs and to perform cellular assays. Briefly, strong anion exchange (SAX) Mega Bond Elute NH_2_ cartridges (1 g) were activated with methanol and equilibrated with 1% acetic acid in water. The ultrasound-assisted solid liquid extract was loaded onto an NH_3_^+^ cartridge and washed with 5 ml of MeOH 1% acetic acid; finally, the glucosinolate fraction was eluted with 10 ml of freshly prepared H_2_O 2% NH_4_OH solution. The purified extract was evaporated to dryness in a vacuum evaporator at 40°C, dissolved in water at a concentration of 1 mg ml^–1^, and filtered with a 22-μm PES filter before cellular assays.

### Qualitative and quantitative analyzes by high-resolution mass spectrometry (HRMS)/MS analysis

Qualitative and quantitative analyzes of GLSs were carried out using an acquity UPLC system coupled with a Xevo G2-XS QT mass spectrometer (Waters Corp., Milford, MA, United States). The mass spectrometer equipped with an electrospray ion source (ESI) was used in negative and positive ionization modes to acquire full-scan MS, and spectra were recorded in the range of 50–1,000 m/z. The source parameters were as follows: electrospray capillary voltage 2 kV, source temperature 150°C, and desolvation temperature 600°C. The cone and desolvation gas flow was 20 and 900 L h^–1^, respectively, and a scan time of 0.3 s was employed. Cone voltage was set at 70 V and source offset at 20. The mass spectrometer was calibrated with 0.5 M sodium formate, and 100 pg μl^–1^of standard leucine-enkkephaline at m/z 554.2615 was infused with the flow of column at 5 μl min^–1^ as the lock mass and acquired for 1 s every 30 s. The total ion current (TIC) used for qualitative analysis was acquired, and an MS/MS spectrum of each compound at different collision energy was acquired and compared to reference standards on which GLS identification was performed. A quantitative analysis was performed using multiple reaction monitoring (MRM) data acquisition mode and by monitoring three characteristic fragments for each target compounds of the [M + H]- ion of glucoarabinin (506.1523 > 442.14, 248.11, and 96.96) glucocamelinin (520.1684 > 456.16, 262.12, and 96.96), and homoglucocamelinin (534.1819 > 470.18, 276.14, and 96.96) and ramping collision energy from 25 to 30 V to produce abundant product ions before detection. In order to quantify the GLS compounds in the extracts, an external standard calibration was conducted six points between 0.01 and 10 μgmL^–1^. Each level was acquired in triplicate. An analysis of variance (ANOVA) was carried out to test the regression curves, and the linear model was found appropriate over the concentration range (R^2^ values > 0.9992). Precision and intraday repeatability were also estimated in all the concentration levels with a coefficient of variation lower than 5%. The results of the quantitative analysis for each analyte were expressed as μg g^–1^of dry matter (DM). The Mass Lynx software (version 4.2) was used for instrument control, data acquisition, and processing.

### Cell cultures

CCD841 (ATCC^®^ CRL-1790™) healthy human mucosa cell lines and CaCo-2 (ATCC^®^ HTB 37™) human colorectal cancer cells were grown in an EMEM medium supplemented with heat inactivated 10% fetal bovine serum (FBS), 2 mM L-glutamine, 1% non-essential amino acids, 100 U ml^–1^penicillin, and 100 μg ml^–1^ streptomycin. E705 (kindly provided by Fondazione IRCCS Istituto Nazionale dei Tumori, Milan, Italy) and SW480 (ATCC^®^ CCL-228™) human colorectal cancer cells were grown in an RPMI 1640 medium supplemented with heat-inactivated 10% FBS, 2 mM L- glutamine, 100 U ml^–1^penicillin, and 100 μg ml^–1^streptomycin. All the cell lines were maintained at 37°C in a humidified 5% CO_2_ incubator. ATCC cell lines were validated by short-tandem repeat profiles that are generated by simultaneous amplification of multiple short-tandem repeat loci and amelogenin (for gender identification). All the reagents for cell cultures were supplied by Lonza (Lonza Group, Basel, Switzerland).

### Viability assay

Cell viability was investigated using an MTT-based *in vitro* toxicology assay kit (Sigma, St. Louis, MO, United States) according to the manufacturer’s protocols. The different cell lines were seeded in 96-well microliter plates at a density of 1 × 10^4^ cells/well, cultured in a complete medium, and treated after 24 h with 400 and 800 μg ml^–1^of glucosinolate purified extract. After 48 h at 37°C, the medium was replaced with a complete medium without phenol red containing 10 μl of 5 mg ml^–1^MTT [3-(4,5-dimethylthiazol-2-yl)-2.5-diphenyltetrazolium bromide]. After 4 h of additional incubation for CCD841 and 2 h for CRC cells lines, formazan crystals were solubilized with 10% Triton X-100 and 0.1 N HCl in isopropanol, and the absorbance was measured at 570 nm using a microplate reader. Cell viabilities were expressed as a percentage against the untreated cell lines used as controls. Two types of statistical analyzes were used using R (version 4.0.0) and GraphPad Prism 8. A general linear model (GLM) was used to evaluate the dose-dependency of the cell lines, while a 2-way ANOVA with Tukey multiple comparison test was conducted to understand differences between the lines at the same concentration of the extract. The significance threshold was set at *p* = 0.05.

### Enzyme assays

To evaluate the effect of glucosinolates on enzymatic activities, CRC cell lines and healthy cell lines were seeded at 1 × 10^6^ cells/100 mm dish and treated for 48 h with the extract at 400 and 800 μg ml^–1^. The cells were rinsed with ice-cold PBS and lysed in 50 mM *Tris*-HCl (pH 7.4), 150 mM NaCl, 5 mM EDTA, 10% glycerol, and 1% NP-40 containing protease inhibitors (1 μM leupeptin, 2 μg ml^–1^aprotinin, 1 μg ml^–1^pepstatin, and 1 mM PMSF). Homogenates were obtained by passing the cells 5 times through a blunt 20-gauge needle fitted to a syringe and then centrifuging them at 15,000 g for 30 min at 4°C. Enzyme activities were assayed on supernatants. Glutathione S-transferase (GST) was measured as reported in Habig (Habig et al., (22)) using 1 mM reduced glutathione (GSH) and 1 mM 1-chloro-2,4-dinitrobenzene (CDNB) as substrates in the presence of a 90-mM potassium phosphate buffer (pH 6.5),and the reaction was monitored at 340 nm. Superoxide dismutase (SOD) was measured using an indirect method according to Vance [Vance et al., (23)]. This technique is based on the ability of SOD to compete with ferricytochrome c for superoxide anions generated by the xanthine oxidase system and, thus, to inhibit the reduction of ferricytochrome c. Briefly, the protein samples were incubated with 0.01 mM ferricytochrome c in 10 mM HEPES-*Tris* (pH 7.5), 0.1 mM EDTA, 0.01 mM xanthine in 1 mM NaOH, and xanthine oxidase at a final concentration of 0.006 U/ml. Under these conditions, one unit of SOD is the amount of enzyme able to yield a 50% decrease in the rate of ferricytochrome c reduction followed at 550 nm. All the assays were performed in triplicate at 25°C with a Jasco V-550 spectrophotometer and analyzed with the Spectra Manager (version 1.33.02) software of Windows. A linear model was chosen for statistical analyzes of enzymatic assays to evaluate differences against a control set at fold = 1. The significance threshold was set at *p* = 0.05.

## Results and discussion

Initially, a chemical characterization of phytochemical compounds in the Camelina seed by-product was performed by UPLC-HRMS. The full ms chromatograms are shown in Supplementary Figure 1, and a list of the tentatively identified phytochemicals numbered according to elution order is shown in Supplementary Table 1. The untarget analysis in negative ion mode allowed for identification of 11 metabolites belonging mainly to two classes, polyphenols and glucosinolates. The results of the qualitative analysis was in accordance with literature data (24–27). However, among all the compounds found in the extract, our attention was focused on glucosinolates. The analysis of chromatogram in full MS and MS/MS mode allowed for us to identify the presence of three main glucosinolates in the extract, which were assigned as glucoarabinin, glucocamelinin, and homoglucocamelinin. The identification was based on retention time and UV and MS/MS spectra and finally confirmed with commercial standards. The results of the qualitative analysis were in accordance with literature data (25, 28, 29). The individual and total GLS contents in *Camelina* sativa seeds were investigated using an international standard method (ISO 9167-1) with some slight modifications and avoiding the desulfation step. A quantitative analysis of the ISO method was carried out using a selective MRM method, and the results showed that the amount of glucoarabinin, glucocamelinin, and homoglucocamelinin was 304.3 ± 62, 403.3 ± 23, and 262.6 ± 87 μg g^–1^DM, respectively.

### Optimization of glucosinolate extraction

#### Selection of solvent composition

**FIGURE 1:**
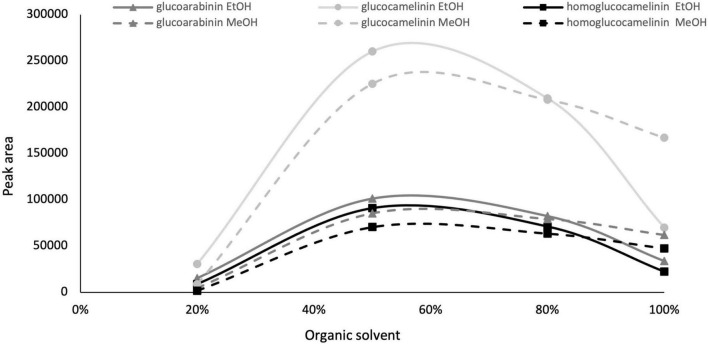
Glucosinolate peak area vs. organic solvent percentage, ethanol (solid line) and methanol (dashed line).

Given the interesting content of GLSs in the extract of *C. sativa* PC, especially considering that the matrix used is an industry by-product, we decided to develop and optimize a green extraction method based on ultrasound-assisted extraction (USAE) to improve extraction yield and reduce the use of chemicals and environmental impact. As commonly reported in the literature, one of the most important parameters that affect the extraction efficiency in USAE process is the composition of the extraction solvent. For this reason, to replace methanol with a green solvent such as EtOH (generally recognized as safe) and to select a solvent composition to be used in the further experimental design, preliminary experiments were carried out by increasing from 0 to 100 the organic solvent percentage (ethanol and methanol) in water, and GLS content was monitored. The other parameters of USAE, namely, solvent volume, extraction cycle, and extraction time were kept constant at 10 ml, 2 cycles, and 5 min, respectively. The results indicate that an extraction solvent with water content higher than 80% formed a mucilaginous agglomerate that makes injection in the chromatographic system impossible. However, as shown in Figure 1, the quantitative trend of monitored GLSs is comparable using both methanol and ethanol. GLS content increases proportionally to the increase in organic solvent from 20 to 60%, but beyond this value it begins to decrease. Based on these results and considering that the behavior of methanol and ethanol was comparable, EtOH in the range of 40 to 80% was selected as extraction solvent in the next optimization step because of its lower environmental impact and toxicity.

#### Response surface design

After the preliminary experiments were carried out to select the organic solvent and its content, a response surface methodology (RSM) was used to maximize the extraction of GLSs and at the same reduce the consumption of the organic solvent. In this study, the influence of the three independent variables (solvent volume, solvent composition, and extraction cycles) on the extraction efficiency of each glucosinolate and on total EtOH consumption was simultaneously evaluated by a Box-Behnken 2 factor interaction design. GLS contents (μgg^–1^DM) were considered as response variable to be maximized and total consumption of EtOH (ml) as variable to be minimized considering that reduction of the organic solvent has a positive influence on the cost of the analysis and on environmental impact. Table 1 reports the total volume of EtOH used and the amount of glucosinolates for each run provided by the CCD used as a response variable. The statistical parameters of the experimental design are summarized in Table 2.

**TABLE 2:** Analysis of variance of the regression model.

	Sum of squares				Mean square			
	Glucoarabinin	Glucocamelinin	Homo glucocamelinin	Tot EtOH	Glucoarabinin	Glucocamelinin	Homo glucocamelinn	Tot EtOH
A: EtOH%	10801.6	66195.2	1826.8	286.8	10801.6	66195.2	1826.8	286.8
B: Volume	769.3	3988.1	293.5	836.4	769.3	3988.1	293.5	836.4
C: Cycles	303.7	749.0	83.3	187.2	303.7	749.0	83.3	187.2
A^2^	7626.5	40568.0	961.2	6.8	7626.5	40568.0	961.2	6.8
B^2^	31.6	4.2	4.9	5.5	31.6	4.2	4.9	5.5
C^2^	66.0	539.9	10.4	5.8	66.0	539.9	10.4	5.8
AB	1073.6	6761.8	137.5	34.2	1073.6	6761.8	137.5	34.2
AC	1218.4	8271.0	135.3	20.3	1218.4	8271.0	135.3	20.3
BC	119.0	1241.9	3.5	109.2	119.0	1241.9	3.5	109.2
Lack of fit	309.1	2120.5	42.0	27.0	103.0	706.8	14.0	9.0
Pure error	97.1	224.9	9.9	0.1	32.4	75.0	3.3	0.0
Total	22415.9	130665.0	3508.2	1519.2				
R^2^	98.2	98.2	98.5	98≅0.2				
Adj. R^2^	95.5	95.5	96.3	95.5				
	**F-value**				***P*-value**			
	**Glucoarabinin**	**Glucocamelinin**	**Homoglucocamelinin**	**Tot EtOH**	**Glucoarabinin**	**Glucocamelinin**	**Homoglucocamelinin**	**Tot EtOH**

A: EtOH%	333.6	882.9	550.8	7821.9	**0.0004^a^**	**0.0001^a^**	**0.0002^a^**	**0.0000^a^**
B: Volume	23.8	53.2	88.5	22811.1	**0.0165^a^**	**0.0053^a^**	**0.0025^a^**	**0.0000^a^**
C: Cycles	9.4	10.0	25.1	5105.8	0.0549	0.0508	**0.0153^a^**	**0.0000^a^**
A^2^	235.5	541.1	289.8	184.4	**0.0006^a^**	**0.0002^a^**	**0.0004^a^**	**0.0009^a^**
B^2^	1.0	0.1	1.5	150.6	0.3958	0.8273	0.3131	**0.0012^a^**
C^2^	2.0	7.2	3.1	157.1	0.2486	0.0748	0.1749	**0.0011^a^**
AB	33.2	90.2	41.5	552.3	**0.0104^a^**	**0.0025^a^**	**0.0076^a^**	**0.0001^a^**
AC	37.6	110.3	40.8	2978.3	**0.0087^a^**	**0.0018^a^**	**0.0078^a^**	**0.0002^a^**
BC	3.7	16.6	1.1	245.4	0.151	**0.0268^a^**	0.3812	**0.0000^a^**
Lack of fit	3.2	9.4	4.2		0.1836	**0.0489^a^**	0.1338	**0.0004^a^**
Pure error								
Total								
R^2^								
Adj. R^2^								

R^2^, quadratic correlation coefficient.

^a^Significant (p < 0.05).

**FIGURE 2:**
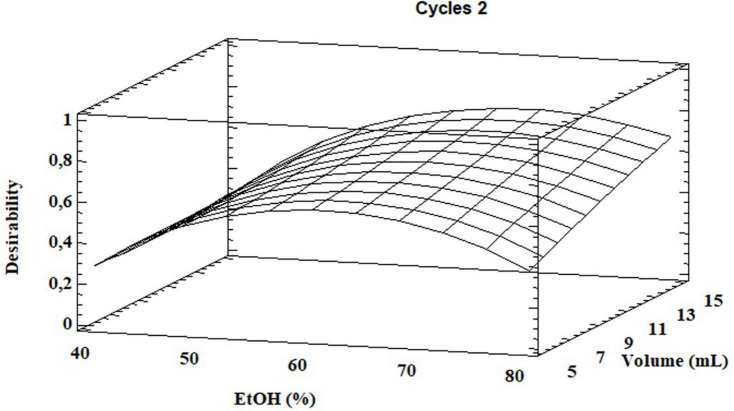
Desirability plot for total glucosinolate extraction as functions of ethanol percentage and total solvent volume.

Based on the results, the model showed a high correlation (R^2^ ≅ 98%), indicating a slight variance of the data and a good prediction of the model with respect to all the considered response variables. Two independent variables, percentage of EtOH and its volume, had a significant influence on both GLS extraction and volume of total EtOH (*p* < 0.05) while the extraction cycles had a significant influence only on the extraction efficiency of homoglucocamelinin and on the volume of EtOH. Moreover, the quadratic effect of multiple parameters as well as the interaction among many parameters was statistically significant (*p* < 0.05) for the response variables considered (Table 2). As shown in the desirability plot (Figure 2), the volume of the extraction solvent linearly influences the desired effect; in fact, by increasing the extraction volume from 5 to 15 ml, there is an increase in desirability. Regarding the percentage of EtOH, the desirable effect increases proportionally to the increase in organic solvent from 40 to ≅70%, but beyond this value it begins to decrease. This result was also in agreement with those obtained in our preliminary results. Finally, the optimized conditions to maximize the extraction of glucosinolate compounds and at same time reduce the consumption of organic solvents were calculated as EtOH 65%, cycles 2, and solvent volume 5 ml. After selecting the optimized extraction conditions to evaluate the improvement in extraction efficiency, these results have been comparted with those obtained using the ISO method. A quantitative analysis of the three glucosinolates was carried out by UPLC-HRMS using the external standard method. The calibration curve of glucosinolates in the concentration range of.1–10 μg ml^–1^ were used to quantify their content in both extracts. The external standard calibration curves for all the analytes provided good linearity within the investigated concentration range with correlation coefficients (R^2^) ranging from 9993 and 9998. The quantitative analysis of the extract obtained by using USAE shows that the content of glucoarabinin, glucocamelinin, and homoglucocamelinin were 1,525.6 ± 53, 3,544.6 ± 209, and 615.5 ± 68 μg g^–1^DM, respectively. This result highlights a huge increase in extraction efficiency for all target compounds, in particular, the recovery of glucoarabinin, glucocamelinin, and homoglucocamelinin increased by 501, 878 and 234%, respectively. These results can be explained by the increased chemical stability of glucosinolates under the milder extraction conditions of the developed USAE method, compared to the ISO procedure which uses methanol at 75°C as extraction solvent. These conditions can cause thermal degradation of glucosinolates as highlighted by some authors (30–33).

### Purification of glucosinolates

Given the high content of GLSs in *Camelina sativa* PC, we decided to develop an efficient protocol based on solid phase extraction (SPE) to obtain a purified extract and test cell activity while preventing other compounds from interfering with results. USA extract 140 mg of was diluted in 10 ml H_2_O and 1% HCOOH and loaded into an SPE column. After the loading, a washing solution using 10 ml H_2_O and 1% HCOOH was passed through the SPE cartridge to remove non-retained compounds, while the GLSs were eluted using 10 ml of H_2_O and 2% NH_4_OH.

**FIGURE 3:**
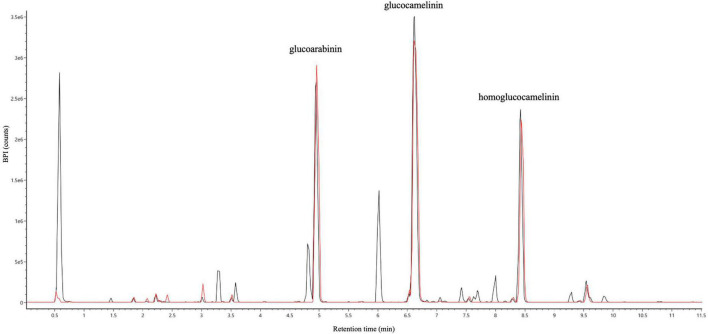
UHPLC full MS chromatograms of ultrasound-assisted solid liquid extraction (USAE) under optimized conditions before (black line) and after (red line) purification by solid phase extraction (SPE).

Both wash and elution fractions were collected, and each one was analyzed by UPLC-HRMS-DAD to detect the presence of GLSs and verify the purity of the SPE extracts. The results of HRMS chromatographic profiling of elution fractions, crude extract, and reference standard compounds are shown in Figure 3. The chromatographic analysis suggests that the developed SPE procedure allowed to selectively purify the GLSs, avoiding losses of compounds of interest in the washing step. In general, the results show that USAE coupled with the SPE procedure allow to obtain a high purity of GLS molecules from *Camelina sativa* PC. An overall balance in the extraction, isolation, and purification processes suggests that approximately 800 mg of purified GLS extract can be recovered from 10 g of PC used.

### Antiproliferative effect of purified glucosinolate extract

**FIGURE 4:**
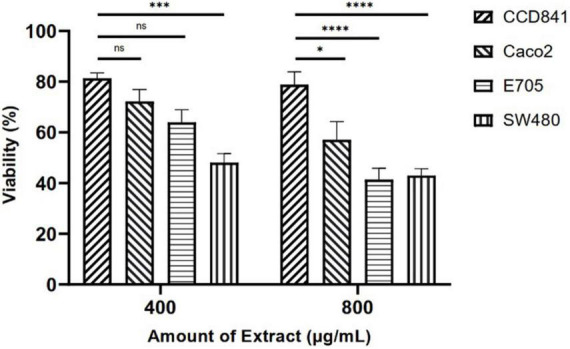
MTT viability assays on four cell lines treated with *Camelina sativa* extracts at two concentrations by 2-way analysis of variance (ANOVA) statistical analysis (ns, not significant, * = *p* < 0.05, ***p < 0.001, and ****p < 0.0001).

**FIGURE 5:**
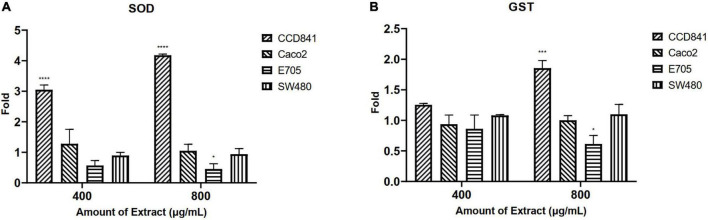
Fold increase in **(A)** superoxide dismutase (SOD) and **(B)** glutathione S-transferase (GST) activity after 48 h of treatment. The statistical analysis used a linear model against a fold control equal to 1 (*p < 0.05, ***p < 0.001, and ****p < 0.0001).

Initially, screening was performed to define the concentrations of purified extract to be used in MTT viability tests. Among the concentrations tested in the range of 100 to 1,000 μg ml^–1^, the experimental results suggested to select a concentration range of 400–800 μg ml^–1^ for further experiments. Four different cell lines were selected to perform MTT assays and evaluate cell viability after a 48-h treatment through mitochondrial activity. The cell lines chosen were healthy colorectal mucosa CCD841 cell lines and three colorectal cancer cell lines with peculiar mutations or behaviors. In particular, CaCo-2 and E705 show no hyperactivating mutations in the KRAS, NRAS, BRAF, and PIK3CA genes, with the E705 cell line carrying a silent mutation in the PIK3CA gene, whereas SW480 carries a hyperactivating mutation in exon 2 of the KRAS gene. The Caco-2 and SW480 cell lines do not respond to cetuximab, a MoAbs against EGFR, while the E705 cell line is sensitive to cetuximab (34). A general linear model analysis on MTT assays demonstrated that the purified extract had a significantly dose-dependent antiproliferative effect on the three tumor cell lines (*p* < 0.001), and that no significant effect (*p* > 0.07) on the healthy cell lines was found, demonstrating specific selectivity against the tumor cell lines. The 2-way ANOVA analysis (Figure 4) using the healthy cell lines as control showed a strong effect at the 800 mg ml^–1^ concentration on the E705 and SW480 cells, where the viability dropped to 40%. Subsequently, to clarify the possible mechanisms involved, two enzymes involved in reactive oxygen species (ROS) detoxification, superoxide dismutase (SOD), and glutathione S-transferase (GST) were assayed. SOD converts the superoxide radical (O_2⋅_^–^) into hydrogen peroxide (H_2_O_2_), while GST is an enzyme that transfers glutathione to xenobiotic substrates. We hypothesize that the extract acts as a stressor element by increasing oxidative metabolism. Upon extract addition, the healthy cells respond physiologically by increasing the activity of the enzymes, but the tumor cell lines do not. Figure 5 shows the increase in both enzymes’ activity: at the highest extract concentration, SOD is increased by 4-fold (*p* < 0.05) and GST by 2-fold (*p* < 0.05) in healthy CCD841 cells. Caco-2 and SW480 maintain the basal level of these enzymes after treatment with both extract concentrations (*p* > 0.05). E705 shows an opposite behavior: the activity of the two enzymes is decreased by 0.5 fold already at 400 μg ml^–1^of extract for SOD (*p* < 0.05) and 800 μg ml^–1^for GST (*p* < 0.05).

## Conclusions

For the first time, a green methodology based on the use of the USAE method with green solvents has been developed to obtain glucosinolates with high purity from *Camelina sativa* seed by-product. The effect of extraction parameters on GLS content in the raw extract and the reduction of organic solvent were optimized using an experimental design. The volume of the solvent and the percentage of ethanol show the main effect on the selected response variables. Under optimized conditions, the procedure allowed for enormous recovery of compounds compared to the ISO method. Based on our results, approximately 800 mg of enriched GLS extract can be obtained from 10 g of *Camelina sativa* PC subjected to USA extraction followed by SPE purification. The purified extract, rich in glucoarabinin, glucocamelinin, and homoglucocamelinin, showed an interesting chemopreventive action against different colorectal cancer cell lines without affecting the healthy cell lines. However, the exact mechanism of action of the purified extract on the tumor cell lines needs further studies to be clarified with a view to developing new sustainable treatments for patients with cancer refractory to conventional chemotherapy. Moreover, the glucosinolate extract can increase the activity of two of the most important enzymes involved in cell defense against oxidative stress, SOD and glutathione S-transferase, thus showing antioxidative properties, which could be exploited in cancer and oxidative stress prevention. The developed method can be considered a suitable green protocol to obtain nutraceutical products with interesting and promising anticancer activities from a natural and cheap food by-product.

## Data availability statement

The original contributions presented in this study are included in the article/Supplementary Material, further inquiries can be directed to the corresponding author.

## Author contributions

ML and LC: conceptualization. SP, DP, RC, and LC: data curation. SP, CG, and RC: formal analysis. SP, CM, MF, and GS: investigation. LM and LC: supervision. LC, PF, and LM: writing – original draft. All authors contributed to the article and approved the submitted version.

## Conflict of interest

The authors declare that the research was conducted in the absence of any commercial or financial relationships that could be construed as a potential conflict of interest.

## Publisher’s note

All claims expressed in this article are solely those of the authors and do not necessarily represent those of their affiliated organizations, or those of the publisher, the editors and the reviewers. Any product that may be evaluated in this article, or claim that may be made by its manufacturer, is not guaranteed or endorsed by the publisher.
