# Supplementary material for: Optimization of ultrasound-assisted extraction of naturally occurring glucosinolates from by-products of Camelina sativa L. and their effect on human colorectal cancer cell line
Source: Front Nutr. 2022 Jul 22;9:901944. doi: 10.3389/fnut.2022.901944 (PMC9355136; doi:10.3389/fnut.2022.901944)
Supplement: Supplementary file 1 [file Data_Sheet_1.docx]

**Supplementary Figures and Tables**

5/6


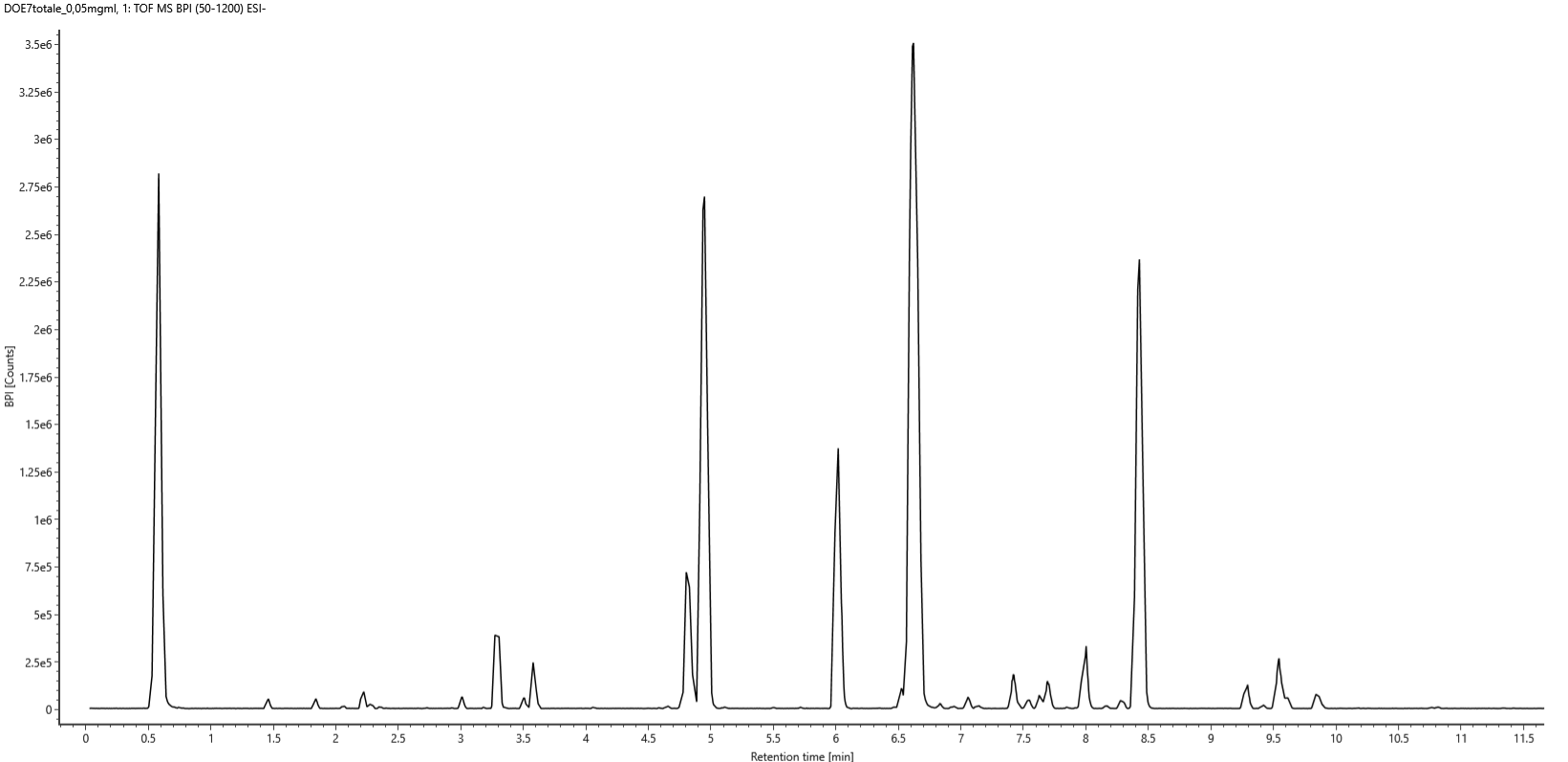


22

7

11

9

8

10

32

4

1

Supplementary Figure 1 UPLC Full MS chromatogram of *Camelina sativa* PC extract acquired in negative ionisation mode

Supplementary Table 1 HRMS and MS/MS data of detected compounds in the *Camelina sativa* PC extract.

| **N°** | **Rt (min)** | **[M-H]^-^** | **formula** | **compounds** | **fragments** | **Δ**  **(ppm)** |
| --- | --- | --- | --- | --- | --- | --- |
| 1 | 3.27 | 451.1246 | C_21_H_24_O_11_ | epicatechin-O-glucoside isomer | 289.0719;  245.0818 | -0.1 |
| 2 | 3.58 | 451.1246 | C_21_H_24_O_11_ | epicatechin-O-glucoside isomer | 289.0719;  245.0818 | 0.1 |
| 3 | 4.95 | 506.1194 | C_17_H_33_NO_10_S_3_ | glucoarabinin | 491.0946, 442.1217; 248.0960; 96.9598 | 0.1 |
| 4 | 6.02 | 741.2214 | C_32_H_38_O_20_ | rutin-2-O-apioside | 609.1474;  301.0337 | 1.4 |
| 5 | 6.62 | 520.1356 | C_18_H_35_NO_10_S_3_ | glucocamelinin | 505.1126; 456.1375, 262.1116; 96.9596 | 1.2 |
| 6 | 6.62 | 609.1468 | C_27_H_30_O_16_ | rutin | 301.0349; 283.0245 | 1.1 |
| 7 | 7.04 | 755.2051 | C_33_H_40_O_20_ | Kaempferol-3-O-gentiobioside-7-O-rhamnoside | 623.1623; 489.1033, 315.0497 | 1.4 |
| 8 | 7.63 | 593.1518 | C_27_H_30_O_15_ | Kaempferol-3-O-neohesperidoside | 285.0401; 151.0033 | 1.1 |
| 9 | 8.00 | 623.1619 | C_28_H_32_O_16_ | Isorhamnetin-3-O-β-rutinoside | 315.0627; 151.0034 | 0.3 |
| 10 | 8.43 | 534.1519 | C_19_H_37_NO_10_S_3_ | homoglucocamelinin | 519.1282; 470.1534; 276.1276; 96.9598 | 3.6 |
| 11 | 9.54 | 623.1627 | C_28_H_32_O_16_ | Tamarixetin-7-O-rutinoside | 209.0451; 108.0207 | -0.1 |
